# Comparative effects of hip capsule repair and cam lesion excision on capsulotomy healing: An in vivo biomechanical and histological analysis

**DOI:** 10.1002/jeo2.70267

**Published:** 2025-05-19

**Authors:** Abbas Aghayev, Burak Duymaz, Selahaddin Aydemir, Pınar Akokay Yılmaz, Gurhan Tukel, Resit Bugra Husemoglu, Onur Gürsan, Onur Hapa

**Affiliations:** ^1^ Department of Orthopedics and Traumatology Dokuz Eylul University Faculty of Medicine İzmir Turkey; ^2^ Department of Orthopedics and Traumatology Kastamonu Research and Training Hospital Kastamonu Turkey; ^3^ Department of Medical Laboratory Techniques Izmır Kavram Vocational School Izmir Turkey; ^4^ Department of Biomechanics Dokuz Eylul University Izmir Turkey

**Keywords:** biomechanical analysis, cam excision, capsular management, capsule repair, femoroacetabular impingement (FAI), histological healing

## Abstract

**Purpose:**

This study evaluates the effects of hip capsule repair and cam lesion excision on capsular healing by assessing biomechanical strength and histological integrity in an in vivo rabbit model.

**Methods:**

An in vivo rabbit model with 80 rabbits was used, where capsulotomy was performed on the right hip of each subject. The rabbits were assigned into four groups: Group 1 (capsulotomy without repair), Group 2 (capsulotomy with capsule repair), Group 3 (capsulotomy + cam resection without repair), Group 4 (capsulotomy + cam resection + capsule repair). Each group was stratified into 4‐week and 8‐week follow‐up subgroups. Biomechanical testing assessed maximum tensile strength, while histological evaluation included semiquantitative grading of collagen arrangement, inflammatory response, osteogenesis, and angiogenesis.

**Results:**

Histological analysis revealed superior healing in the capsule repair + cam resection group (Group 4) compared to the unrepaired capsulotomy group (Group 1) (*p* = 0.01). Biomechanical testing demonstrated that capsule repair (Group 2) improved strength over unrepaired capsulotomy (135.2 N vs. 111.9 N, *p* = 0.03). Cam resection alone (Group 3) resulted in significantly higher strength than unrepaired capsulotomy (163.2 N vs. 111.9 N, *p* = 0.01). The combination of cam resection and capsule repair (Group 4) demonstrated superior strength, outperforming capsule repair alone (176 N vs. 135.2 N, *p* = 0.01). At 8 weeks, the capsule repair + cam resection group (Group 4a) showed significantly enhanced biomechanical strength compared to the unrepaired capsulotomy group (Group 1a) (181.6 N vs. 120.9 N, *p* = 0.001) and capsule repair alone (Group 2a) (181.6 N vs. 125.8 N, *p* = 0.01).

**Conclusion:**

Our findings indicate that cam resection, particularly when combined with capsule repair, significantly improves biomechanical strength and enhances the healing process of the capsule. These findings offer practical guidance for optimising surgical strategies to enhance patient outcomes and long‐term joint function.

**Level of Evidence:**

Level III, experimental therapeutic study (prospective and controlled).

**Abbreviations**
ANOVA: analysis of variance
FAI: femoroacetabular impingement
H&E: hematoxylin and eosin
MNL: mononuclear leucocyte
N: Newton
*p* value: probability value
SPSS: Statistical Package for the Social Sciences

## INTRODUCTION

Femoroacetabular impingement (FAI) is a significant orthopaedic condition that results in hip pain and functional limitations, predominantly affecting young and active individuals [4]. This condition arises from abnormal contact between the femoral head and the acetabulum, leading to labral and cartilage damage [16]. While hip arthroscopy is widely recognised as the gold standard for FAI treatment, there remains a lack of consensus regarding the optimal management of capsular, labral injuries, particularly in terms of capsulotomy repair and cam lesion excision [4, 10]. This study aims to address these gaps by evaluating the biomechanical and histological outcomes of combining capsule repair with cam excision, providing insights that may guide surgical strategies to optimize patient outcomes.

The existing literature presents conflicting findings regarding the outcomes of capsule closure versus leaving it open following capsulotomy and cam resection [6, 10]. While some studies suggest that capsule closure provides better biomechanical stability and improves clinical outcomes, others argue that this procedure is unnecessary and that open capsules can heal over time [6, 10, 13]. Furthermore, in vivo studies exploring the histological changes influencing the healing process in repaired versus unrepaired capsules after capsulotomy remain limited [13].

This study was designed as a significant step toward understanding the impact of capsular management in the treatment of femoroacetabular impingement (FAI). Despite advancements in FAI treatment, there remains limited knowledge regarding the biomechanical and histological effects of capsular repair versus unrepaired capsules following capsulotomy [13]. The primary objective of this study was to evaluate these differences and address the gap in understanding how capsular management strategies influence healing. Additionally, this study explores the effects of additional surgical interventions, such as the removal of cam‐type lesions, which remain underreported in the current literature, particularly in the context of their combined effect with capsular repair. Ng et al. [17] emphasised the biomechanical implications of cam lesion excision, yet the interplay between cam resection and capsular repair on healing outcomes remains poorly understood.

To achieve these objectives, an in vivo rabbit model was utilized, marking the first study to employ this model for investigating the effects of capsulotomy and cam excision with or without capsule repair. This controlled experimental environment enabled precise biomechanical and histological analyses, which are challenging to achieve in clinical settings. By combining these evaluations, the study provides a comprehensive perspective that extends beyond prior research, offering novel insights into the structural and functional outcomes of capsular management strategies. This innovative approach aims to address critical gaps in the existing literature and contribute valuable evidence to guide future clinical practices.

Our hypothesis posits that groups undergoing capsule repair after capsulotomy and cam resection would exhibit superior biomechanical durability and histological healing. In contrast, in groups where the capsule was left unrepaired and cam lesions were removed, we anticipate limited healing due to adhesions. By addressing these specific gaps in the literature and offering evidence‐based insights, this study aims to significantly contribute to the understanding of capsular management strategies in FAI treatment, ultimately guiding clinical practice toward optimising surgical outcomes for better patient care.

## METHODS

In this study, we employed an in vivo rabbit model for biomechanical and histological analyses following capsulotomy and cam resection with or without capsular repair. This methodology was selected due to its ability to closely simulate the biomechanical environment of the human hip joint while maintaining a controlled experimental setup [11, 12]. The use of an animal model allowed for standardised surgical procedures and consistent follow‐up periods, minimising variability. Specifically, the rabbit model was chosen for its well‐documented use in joint studies and its capacity to provide sufficient sample sizes for meaningful statistical comparisons [11, 22]. The methodology integrates both biomechanical and histological evaluations to comprehensively assess the effects of different capsular management strategies, bridging a critical gap in the existing literature.

The sample size of 10 specimens per group was determined based on similar studies in the literature examining capsular repair and cam resection [11, 12, 22]. This selection ensures consistency with prior research while allowing for meaningful comparisons. Maintaining this sample size helps strengthen the study's reliability and contributes valuable insights into the role of capsular management strategies in FAI treatment.

The study protocol was approved by the relevant ethical review board. All experimental procedures were conducted in compliance with the International Guide for the Care and Use of Laboratory Animals. Necessary measures were taken to ensure the highest level of animal welfare during and after surgical interventions.

A total of 80 female New Zealand rabbits, each weighing between 3000 and 3500 g, were used in the study. The rabbits were randomly divided into four main groups, each further subdivided into two subgroups based on follow‐up durations of 4 weeks and 8 weeks. Each subgroup consisted of 10 rabbits (Table 1). The right hip of each rabbit underwent surgical intervention, while the left hip served as the control. All animals were maintained under standardised laboratory conditions with consistent feeding and care protocols.

**Table 1:** Group allocation.

Group	Time	Procedure
Group 1	4 weeks	Capsulotomy (*n*:10)
Group 1a	8 weeks	Capsulotomy (*n*:10)
Group 2	4 weeks	Capsulotomy + Repair (*n*:10)
Group 2a	8 weeks	Capsulotomy + Repair (*n*:10)
Group 3	4 weeks	Capsulotomy + Cam Resection (*n*:10)
Group 3a	8 weeks	Capsulotomy + Cam Resection (*n*:10)
Group 4	4 weeks	Capsulotomy + Cam Resection and Repair (*n*:10)
Group 4a	8 weeks	Capsulotomy + Cam Resection and Repair (*n*:10)

### Surgical procedures

The surgical procedures were performed under general anaesthesia in sterile surgical conditions. The rabbits were anaesthetised using an intramuscular combination of ketamine (50 mg/kg) and xylazine (5 mg/kg) and the depth of anaesthesia was continuously monitored during the operation. To maintain respiratory function, the rabbits were ventilated throughout the procedure and their body temperature was stabilised using a heating pad.

All surgical procedures were performed by a senior surgeon with over 15 years of experience in hip arthroscopy and femoroacetabular impingement (FAI) treatment. A standard lateral incision was made on the right hip of each rabbit to access the surgical field. The following steps were performed sequentially.

**Figure 1:**
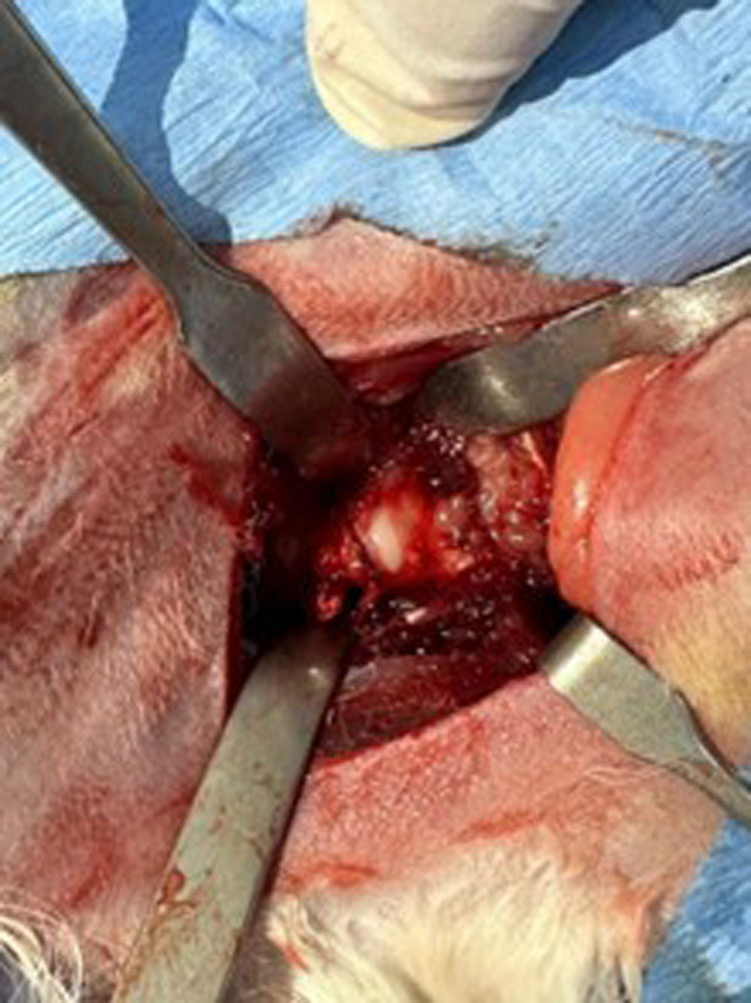
The hip capsule and capsulotomy.

1. Access to the joint capsule and capsulotomy:

After making a skin incision, the retinaculum and the superficial aponeurosis of the anterolateral hip muscles were carefully opened. The anterolateral muscles were then split to expose the hip joint. A 2 cm long transverse capsulotomy was performed anterior to the femoral head, 1 cm lateral to the labrum, using a technique similar to interportal capsulotomy to fully expose the femoral head and acetabulum (Figure 1). Consistency was maintained by using the same anatomical reference points in all procedures, whether the capsule was left open or repaired.

**Figure 2:**
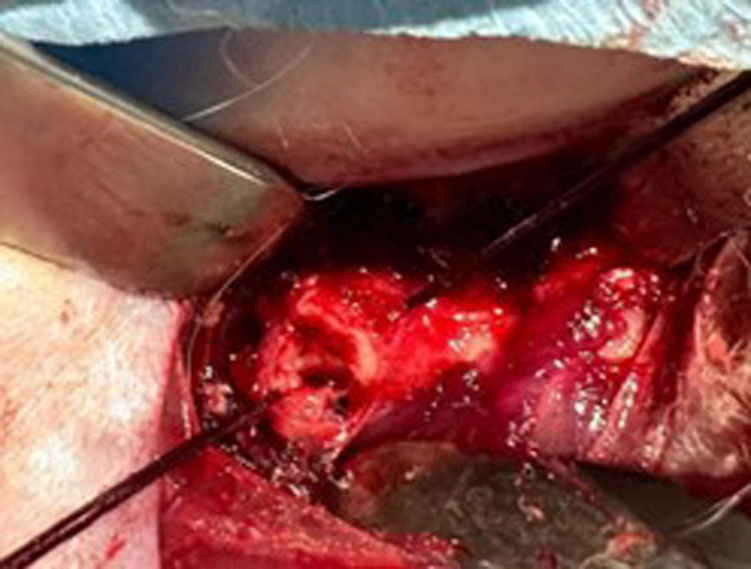
Cam resection.

2. Removal of femoral cam lesion:

Abnormal bony protrusions (cam lesions) located at the femoral head‐neck junction were carefully resected using a motorised burr (Figure 2). During this procedure, special care was taken to avoid damage to the joint labrum and cartilage. After completing the resection, the site was inspected to ensure that no sharp edges remained.

3. Management of capsulotomy:
  Following capsulotomy, the rabbits were randomly assigned to four groups based on the intervention applied. Group 1 (Open Capsule) underwent capsulotomy without subsequent repair, allowing natural healing to take place. Group 2 (Capsule Repair) also underwent capsulotomy; however, the capsule was meticulously repaired using 4‐0 absorbable Vicryl sutures to maintain anatomical integrity. Group 3 (Open capsule + Cam Excision) underwent capsulotomy without repair, but additionally femoral cam excision was performed to evaluate its isolated effect on healing. Group 4 (Cam Excision + Capsule Repair) received both femoral cam excision and capsulotomy, followed by meticulous capsular repair using absorbable sutures.
4. Closure of the surgical site:
  Upon completing all surgical procedures, the muscle and skin layers were closed in layers. Absorbable suture material was used for the muscle layer, while nylon sutures were used for the skin closure. The surgical site was sterilised, analgesic and antibiotic treatments were administered.
5. Postoperative care:
  Postoperative pain management was provided with meloxicam (0.2 mg/kg). To minimise the risk of infection, intramuscular cefazolin (25 mg/kg) was administered. The rabbits were closely monitored for the first 48 h post‐surgery and any complications were promptly addressed.
6. Collection of joint samples:
  At the end of the designated follow‐up periods (4 and 8 weeks), the rabbits were sacrificed in accordance with ethical procedures. Hip joints were collected for biomechanical and histological analyses. For each group, six animals were used for biomechanical testing and four animals were used for histological evaluation at both the 4‐week and 8‐week time points.

### Biomechanical analysis

**Figure 3:**
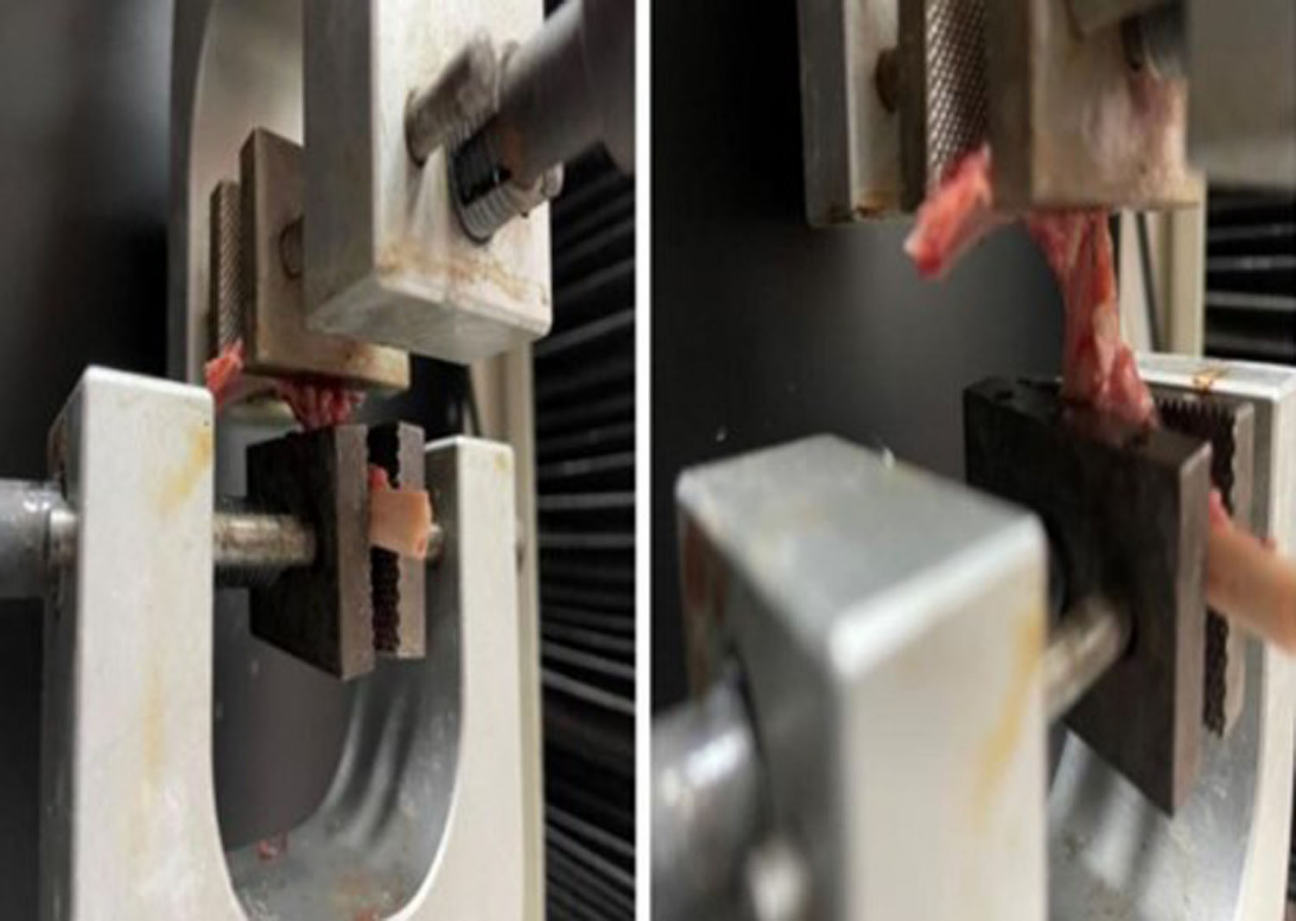
Biomechanical test setup. This figure demonstrates the placement of the femur and acetabulum in the Shimadzu AG‐IS 10 kN device, ensuring precise alignment for tensile testing.

To evaluate the biomechanical durability of the surgical procedures, rabbit hip joints were tested using the Shimadzu AG‐IS 10 kN device, following the protocol described by Weber et al. [24]. This method has been previously validated for assessing capsular biomechanical strength under controlled conditions. The testing setup applied a standardized axial traction force to measure the maximum force required for capsular failure, which is a widely accepted approach in biomechanical studies. The ilium, ischium, and femur were trimmed to appropriate dimensions and positioned horizontally along their long axis between the metal grips of the Shimadzu AG‐IS device (Figure 3). Both ends were securely fixed, leaving the hip joint free to move. This allowed biomechanical testing to align with the natural orientation of the capsule fibres. The samples were subjected to a preload of 50 Newton (N), followed by tensile loading at a speed of 0.1 mm/s (approximately 1.25%/s) until failure. The failure force(maximum force the joint capsule could withstand before rupture) values of each specimen were recorded for biomechanical analysis.

### Histological analysis

Histological analysis was conducted by a histology and embryology professor with over 10 years of professional experience, currently working at a university hospital. Following tissue collection, the samples were immediately fixed in a 10% neutral formalin solution for 48 h to ensure optimal preservation of tissue morphology. After fixation, the specimens underwent a dehydration process using ascending concentrations of ethanol before being embedded in paraffin blocks. Thin sections, ranging from 4 to 6 µm, were obtained using a microtome and subsequently prepared for histological examination.

Histological sections were stained using three different techniques to evaluate various tissue parameters. Hematoxylin and eosin (H&E) staining was utilized to assess general tissue morphology and inflammatory cell infiltration. Masson's Trichrome staining was performed to evaluate collagen fibril organisation and connective tissue formation, while Safranin O staining was used to analyse glycosaminoglycan content within the cartilage matrix.

The stained sections were examined under a light microscope (Olympus BX53, Olympus Corporation, Tokyo, Japan) at 10x, 40x, and 100x magnifications to assess histological parameters. These included collagen fibril organisation (regularity and density of the collagen structure), new bone formation (osteoblast activity and areas of mineralised bone formation), inflammatory cell infiltration (density of lymphocytes, macrophages, and neutrophils) and vascularisation (presence and density of newly formed blood vessels). These parameters were systematically evaluated to determine the healing response following different capsular management strategies.

### Histological scoring

**Figure 4:**
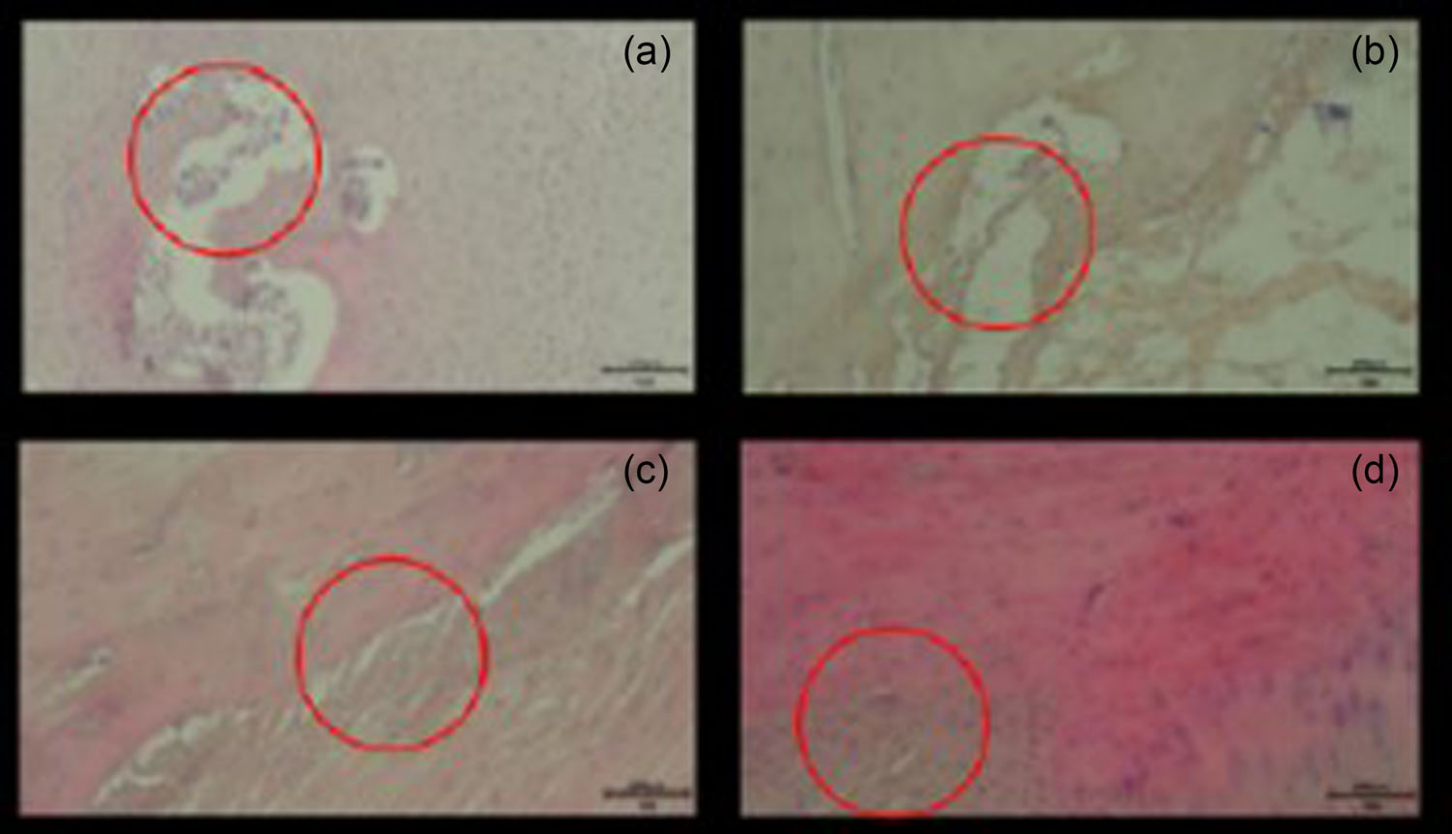
Scoring the extent of healing (a: 0 point, b: 1 point, c: 2 points d: 3 points).

The extent of tissue healing was evaluated using a standardized grading system [1, 19]. A score of 0 indicated no healing, while a score of 1 represented minimal healing. Moderate healing was assigned a score of 2, reflecting an intermediate level between minimal and robust healing. A score of 3 denoted strong and uniformly distributed healing throughout the section (Figure 4). To ensure consistency, all sections were independently evaluated by a blinded histologist and the average score was calculated [19].

### Imaging and analysis

Digital images of histological sections were captured using a digital microscope (Olympus DP74). Quantitative and semi‐quantitative analyses were performed using ImageJ software. Data from each group were statistically compared to identify differences.

### Statistical analysis

The biomechanical and histological data obtained were analysed using IBM SPSS Statistics for Windows (Version 26.0). The normality of data distribution was assessed using the Shapiro–Wilk test. For data that met the assumption of normality, one‐way analysis of variance (ANOVA) was applied to compare differences between groups. When significant differences were detected through ANOVA, Tukey's post hoc test was used for pairwise comparisons. If the assumption of normality was not met, the Kruskal–Wallis test was employed for analysis, and significant results were further evaluated with the Mann–Whitney *U* test for pairwise comparisons.

Data were presented as mean ± standard deviation for normally distributed parameters. A *p*‐value of <0.05 was considered statistically significant for all analyses.

## RESULTS

### Biomechanical results

Significant differences in maximum force values were observed among the groups (Table 2). The cam resection + capsule repair group (Group 4; 176.0 N) and its 8‐week follow‐up counterpart (Group 4 A; 181.6 N) demonstrated the highest maximum force values. A significant difference was noted between the capsule repair group (Group 2; 135.2 N) and the unrepaired capsulotomy group (Group 1; 111.9 N) (*p* = 0.03), as well as between the cam resection without repair group (Group 3; 163.2 N) and the unrepaired capsulotomy group (Group 1; 111.9 N) (*p* = 0.01). Additionally, the cam resection + capsule repair group (Group 4; 176.0 N) showed significantly higher maximum force values compared to the capsule repair alone group (Group 2; 135.2 N) (*p* = 0.01).

**Table 2:** Maximum mean force (N) table for each group after biomechanical tests.

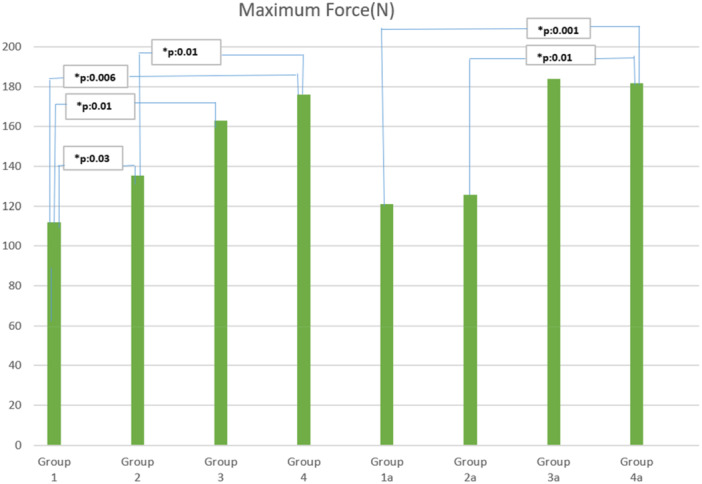

In the 8‐week follow‐up comparisons, the cam resection + capsule repair group (Group 4A; 181.6 N) demonstrated superior biomechanical strength compared to the unrepaired capsulotomy group (Group 1A; 120.9 N) (*p* = 0.001) and the capsule repair group (Group 2A; 125.8 N) (*p* = 0.01). No significant difference was detected between the 4‐week (Group 4; 176.0 N) and 8‐week (Group 4A; 181.6 N) cam resection + capsule repair groups (*p* > 0.05), although Group 4A consistently showed slightly higher values across the comparisons.

### Histological results

In the analysis of tissue samples (Table 3) collected from subjects left to heal for 4 weeks, untreated groups (Group 1 and Group 3) demonstrated mononuclear leucocyte (MNL) infiltration and irregularly aligned collagen fibres. While there was evidence of cell proliferation in the damaged regions, the repair process appeared incomplete. Both groups exhibited a sparse extracellular matrix, indicating limited tissue organization.

**Table 3:** Histological scores.

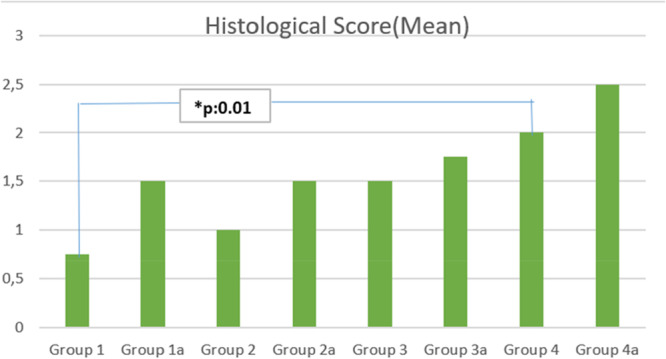

In contrast, the analysis of Groups 2 and 4 revealed the absence of MNL infiltration in the damaged areas. Notably, Group 4 showed a more organised arrangement of collagen fibres compared to Group 2, suggesting a more advanced stage of tissue repair.

For tissue samples collected after 8 weeks of healing, the repair process appeared more advanced compared to the 4‐week groups. The cam excision and repair group (Group 4) exhibited closure of the damaged regions with proliferative cells, demonstrating improved tissue organization and healing compared to other groups.

Significant differences in histological scores were observed between Group 1 (unrepaired capsulotomy) and Group 4 (cam resection + capsule repair) (*p* = 0.01), favoring Group 4. The mean histological scores for these groups were 1.0 ± 0.5 and 2.5 ± 0.6, respectively indicating superior healing in the capsule repair + cam resection group.

Comparisons between Group 1 and Group 3 (cam resection without repair) (*p* = 0.09), Group 1A (8‐week unrepaired capsulotomy) and Group 4A (8‐week cam resection with capsule repair) (*p* = 0.06), and Group 2A (8‐week capsule repair) and Group 4A (*p* = 0.06) yielded *p*‐values close to significance. The mean histological scores for these groups were Group 1: 1.0 ± 0.5, Group 3: 1.8 ± 0.4, Group 1A: 1.3 ± 0.6, Group 4A: 2.7 ± 0.5, Group 2A: 2.0 ± 0.5, further supporting the trend towards improved healing in the cam resection with capsule repair groups. Additionally, the comparison between Group 4 (4‐week follow‐up) and Group 4A (8‐week follow‐up) (*p* = 0.09) suggests that the healing process continues to improve over time, with mean histological scores increasing from 2.5 ± 0.6 to 2.7 ± 0.5 over the 8‐week period.

**Figure 5:**
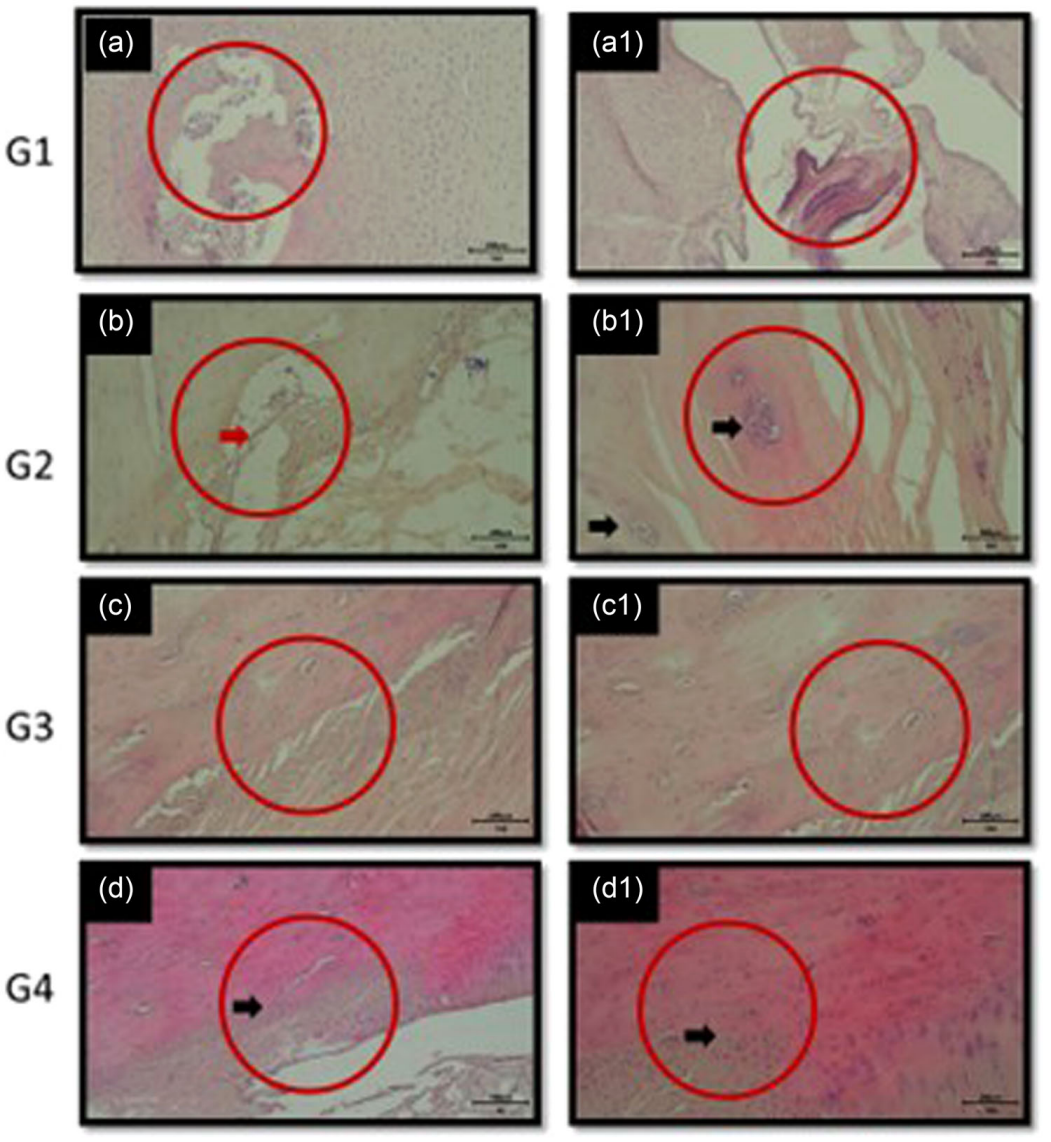
Histological sections.

Histological sections from the hip joint capsule stained with hematoxylin and eosin (H&E). The sections on the left (a, b, c, d) represent the 4‐week results, while those on the right (a1, b1, c1, d1) depict the 8‐week results, all under 10X magnification (Figure 5).

- a and a1: Images from groups that underwent only capsulotomy (4 and 8 weeks).
- b and b1: Images from groups where the capsule was repaired after capsulotomy (4 and 8 weeks).
- c and c1: Images from groups that underwent cam excision (4 and 8 weeks).
- d and d1: Images from groups where the capsule was repaired following cam excision (4 and 8 weeks).

Scores represent the semi‐quantitative evaluation for each specimen mean [1, 8, 19].

Key evidence supporting the impact of cam excision on biomechanical and histological outcomes includes several notable findings.

1. Biomechanical comparisons between the 8‐week capsule repair group (Group 2A) and the 8‐week cam resection + capsule repair group (Group 4 A) (*p* = 0.06), as well as between the 8‐week cam resection without repair group (Group 3A) and Group 4A (*p* = 0.09), demonstrated a trend toward significance, suggesting that cam excision may contribute to improved biomechanical stability.
2. Histological analysis also revealed near‐significant differences between the unrepaired capsulotomy group (Group 1) and the cam resection without repair group (Group 3) (*p* = 0.09), whereas no significant difference was observed between Group 1 and the capsule repair group (Group 2), indicating that cam excision alone may have a more pronounced effect on tissue remodelling compared to capsule repair alone.
3. Additionally, biomechanical strength was significantly greater in the capsule repair group (Group 2) compared to the unrepaired capsulotomy group (Group 1) (*p* = 0.03), confirming the role of capsule repair in improving structural integrity. However, Group 3 (cam resection without repair) exhibited even higher biomechanical strength compared to Group 1 (*p* = 0.01), further emphasising the beneficial impact of cam excision on mechanical stability.
4. Finally, a significant difference was observed between Group 2A (8‐week capsule repair) and Group 4A (8‐week cam resection + capsule repair) (*p* = 0.01), reinforcing the finding that cam excision enhances biomechanical outcomes when combined with capsule repair. In contrast, no significant difference was found between Group 3A (8‐week cam resection without repair) and Group 4A, suggesting that capsule repair may not provide additional biomechanical benefits when cam excision has already been performed. These findings highlight the critical role of cam excision as both a standalone intervention and a synergistic factor when combined with capsule repair, leading to superior mechanical and histological healing outcomes.

## DISCUSSION

This study highlights that combining capsular repair with cam lesion excision results in superior biomechanical and histological healing outcomes, significantly enhancing the stability and repair of the joint capsule in FAI treatment. Cam excision alone demonstrated a stronger impact on biomechanical integrity compared to capsular repair, as shown by significant differences in maximum tensile strength and healing scores. However, when cam excision and capsular repair were combined, the synergistic effect yielded the most robust healing, suggesting that these interventions complement each other in optimising joint function. This finding supports the notion that addressing both structural deformities and capsular integrity is key to achieving long‐term clinical success, aligning with emerging evidence that underscores the role of comprehensive capsular management strategies in improving postoperative outcomes.

The literature presents varying perspectives on the necessity of capsule repair following capsulotomy. Domb et al. [5] emphasised the positive impact of capsule repair on clinical outcomes, whereas Strickland et al. [20] found no significant long‐term differences in outcomes for repaired and unrepaired interportal capsulotomies. Bech et al. [2], in their meta‐analysis, reported no significant differences in clinical outcomes between patients with and without capsule repair, emphasising the need to consider factors such as capsulotomy size, suture technique, and suture count in future studies. Although capsule repair showed promising results in this study, some findings align with the limitations highlighted in the existing literature.

Nguyen et al. [18] demonstrated that morphological changes in the hip capsule, such as variations in capsule thickness, were associated with clinical outcomes including pain, functionality, and return to sports in patients without capsule repair. However, their study also indicated that the capsule could heal without surgical repair, and these morphological changes had a limited impact on long‐term clinical outcomes.

Kraeutler et al. [11] also observed that groups with capsule repair demonstrated superior outcomes at 6 weeks postoperatively; however, these differences disappeared by the 24‐week follow‐up. This suggests that while capsule repair may be more effective in the early stages of recovery, its long‐term benefits could be limited.

Yang et al. [25], in their retrospective study involving 59 hips, analysed preoperative and postoperative MRI changes following routine capsular closure. At a 2‐year follow‐up, patients with fully healed capsules (control group) were compared to those with unhealed or partially healed capsules. The majority of hip capsules were fully healed; however, the study group showed significantly lower hip scores and a reduced likelihood of achieving an acceptable symptomatic state compared to the control group. This demonstrated that capsule repair preserves anterior capsule integrity and thickness following hip arthroscopy, resulting in fewer capsular defects and more effective healing. Similarly Gao et al. [7], in their retrospective study, evaluated preoperative and postoperative MRI images at a minimum follow‐up of six months conducted on 194 patients, the study found that patients without capsular defects achieved better clinical outcomes.

Literature continues to emphasise the critical role of capsular integrity in achieving optimal outcomes following femoroacetabular impingement (FAI) surgery [23]. Kunze et al. [14] demonstrated that capsular closure significantly increases the likelihood of achieving the minimal clinically important difference (MCID) in hip‐specific functional scores, such as the modified Harris Hip Score (mHHS), compared to capsulotomy without repair. This finding highlights the biomechanical importance of capsular management during hip arthroscopy. Similarly, Liu et al. [15] performed a meta‐analysis and concluded that while capsular closure does not universally guarantee superior outcomes in all functional measures, it significantly influences specific scores like the Hip Outcome Score–Activities of Daily Living (HOS‐ADL), providing evidence for its clinical relevance. Furthermore, recent clinical studies have demonstrated that complete capsular closure after arthroscopic FAI treatment and labral repair leads to significantly improved long‐term functional outcomes compared to leaving the capsule open. Patients who underwent full capsular closure showed superior improvements in sports‐specific outcome measures and daily living activities, reinforcing the importance of preserving capsular integrity during hip arthroscopy [21].

Similarly, in our study, hips with histologically improved capsules demonstrated greater biomechanical maximum force, further highlighting the importance of capsular integrity in promoting effective healing.

However, Strickland et al. [20] observed no significant differences in long‐term healing rates between repaired and unrepaired bilateral hip arthroscopies. Additionally, Hapa et al. [9] noted high rates of spontaneous capsular healing (81%) in patients without repair, suggesting that capsule repair may not be the single factor related to healing, other surgical components like cam excision may also be determinant as proven at the present study.

The primary limitation of this study is the use of an animal model. The anatomical and biomechanical differences between rabbits and humans limit the direct applicability of the findings to clinical practice. Additionally, the relatively small sample size is another limitation, which may affect the generalisability of the results. Furthermore, this study did not assess the impact of different suture materials, surgical techniques, or other variables that could potentially influence the healing process. Another important limitation is that, in clinical practice, capsulotomy is rarely performed without concurrent cam resection. However, in this study, there were groups where capsulotomy was performed without cam excision, which may limit the direct clinical relevance of the findings. Additionally, the biomechanical analysis focused on capsular strength rather than overall hip joint stability. While this provides valuable insights into tissue healing, it does not fully represent the functional stability of the hip, which is a key factor in clinical decision‐making. Beck et al. [3] demonstrated that adhesions between the joint capsule and the resected femoral neck contribute to groin pain and that pain is relieved by the removal of these adhesions. However, the presence or effect of these adhesions was not evaluated in our study.

This study demonstrates that the combination of cam excision and capsule repair yields the most favourable outcomes in terms of capsular healing. Cam excision, in particular, had a more pronounced effect on enhancing capsular healing compared to capsule repair alone. However, capsule repair contributed significantly to biomechanical stability and tissue regeneration. These findings suggest that different patient populations may benefit from distinct surgical approaches, highlighting the importance of individualised treatment strategies. For instance, patients with hip microinstability may benefit more from capsule repair, whereas muscular patients or those with early arthritic changes and preexisting joint stiffness may achieve better outcomes without repair. Understanding these differences is crucial for optimising patient selection and improving surgical decision‐making in FAI treatment.

Future research should further investigate the role of additional interventions, such as cam excision, in promoting capsular healing, with a particular focus on understanding the underlying biochemical mechanisms, including growth factor release. Additionally, correlating biomechanical and histological findings with long‐term clinical outcomes in human patients would provide valuable insights into the real‐world effectiveness of these interventions. Procedures such as microfracturing at the head‐neck junction could also be explored as potential strategies to enhance capsule healing and address symptomatic capsular defects. Expanding clinical studies to evaluate which patient subgroups respond best to capsular repair or non‐repair approaches could further refine evidence‐based surgical guidelines for FAI management.

## CONCLUSION

This study demonstrated that the combination of capsular repair and cam lesion resection in the treatment of FAI provides superior biomechanical stability and histological improvement. While cam resection alone provided significant improvement, a synergistic effect was achieved when combined with capsular repair, with the strongest results. The findings emphasise that focusing surgical strategies on both preservation of capsular integrity and correction of structural deformities is critical for long‐term clinical success. Future studies may further improve treatment outcomes by further investigating the biochemical mechanisms underlying these combined interventions.

## AUTHOR CONTRIBUTIONS

*Concept*: Abbas Aghayev and Onur Hapa. *Design*: Burak Duymaz, Selahaddin Aydemir, and Onur Hapa. *Supervision*: Onur Gürsan and Onur Hapa. *Materials*: Gurhan Tukel, Resit Bugra Husemoglu, and Pınar Akokay Yılmaz. *Data collection*: Resit Bugra Husemoglu and Pınar Akokay Yılmaz. *Analysis*: Resit Bugra Husemoglu and Pınar Akokay Yılmaz. *Literature search*: Abbas Aghayev, Gurhan Tukel, and Selahaddin Aydemir. *Writing*: Burak Duymaz. Corresponding Author: Burak Duymaz; Review: Burak Duymaz, Onur Gürsan, and Onur Hapa. All authors approved the final version of the manuscript.

## CONFLICT OF INTEREST STATEMENT

The authors declare no conflicts of interest.

## ETHICS STATEMENT

This study received ethical approval from Dokuz Eylul University Animal Experiments Local Ethics Committee (Approval No: 13/2022). All procedures followed international guidelines for animal research. Efforts were made to minimise animal suffering through the use of anaesthesia, analgesia, and proper postoperative care. Animals were housed in standard laboratory conditions and humanely euthanized at the end of the study.

## Data Availability

The data that support the findings of this study are available from the corresponding author upon reasonable request.
